# Neighborhood perception and depressive symptoms: the ELSI-Brasil national study, 2015–2016

**DOI:** 10.11606/s1518-8787.2025059006948

**Published:** 2025-12-08

**Authors:** Pablo Cardozo Roccon, Amanda Cristina de Souza Andrade, Débora Moraes Coelho, Bruno de Souza Moreira, Luciana de Souza Braga, Maria Fernanda Lima-Costa, Waleska Teixeira Caiaffa

**Affiliations:** I Universidade Federal de Minas Gerais. Faculdade de Medicina. Departamento de Medicina Preventiva e Social. Belo Horizonte, MG, Brazil; II Universidade Federal de Minas Gerais. Observatório de Saúde Urbana de Belo Horizonte. Belo Horizonte, MG, Brazil; III Universidade Federal de Mato Grosso. Instituto de Saúde Coletiva. Programa de Pós-Graduação em Saúde Coletiva. Cuiabá, MT, Brazil; IV Fundação Oswaldo Cruz. Instituto René Rachou. Belo Horizonte, MG, Brazil; V Núcleo de Estudos em Saúde Pública e Envelhecimento. Belo Horizonte, MG, Brazil; VI Universidade Federal de Minas Gerais. Faculdade de Medicina. Programa de Pós-Graduação em Saúde Pública. Belo Horizonte, MG, Brazil

**Keywords:** Depressive Symptoms, Neighborhood Environment, Neighborhood Effects, Perception, Middle-Aged Person, Older adult

## Abstract

**OBJECTIVE:**

To investigate the association between depressive symptoms and neighborhood perception among older adults living in urban areas.

**METHODS:**

This cross-sectional study included 7,115 individuals (≥ 50 years) from the baseline (2015–2016) of the *Estudo Longitudinal da Saúde dos Idosos Brasileiros* (ELSI-Brazil – Brazilian Longitudinal Study of Aging) who lived in urban areas and had self-reported responses (no proxy respondents), with complete data on the outcome. The outcome was assessed using the 8-item Center for Epidemiologic Studies–Depression Scale (CES-D-8) (cutoff ≥ 4), and the exposure variable was neighborhood perception related to participants’ area of residence. Univariable and multivariable Poisson regression analyses were performed, with each exposure variable adjusted for sociodemographic characteristics and health conditions.

**RESULTS:**

The prevalence of depressive symptoms was 34.9%, being 43.8% among women and 24.5% among men. In the multivariable analysis, urban mobility issues, noise pollution, physical disorder, violence, lack of neighborhood pleasantness, low social cohesion, and perceived insecurity were significantly associated with a higher prevalence of depressive symptoms in the overall sample and among women. Among men, all variables except perceived insecurity were also significantly associated with higher prevalence of depressive symptoms.

**CONCLUSION:**

The findings highlight the importance of intersectoral action across health, urban mobility, public safety, and urban planning policies to promote mental health among older adults living in urban areas.

## INTRODUCTION

Depressive symptoms can have a significant impact on the health, quality of life, and psychosocial well-being of older adults^1^. According to meta-analyses, 35.1% of older adults worldwide experience depressive symptoms^2^; in Brazil, the prevalence is 21.0%^3^.

Studies have shown that the urban environment—natural, built, social, and economic —can either promote health or contribute to inequities among residents^4^. Social and physical characteristics of the neighborhood can generate daily doses of stress, increasing the prevalence of depressive symptoms among older adults^5^.

Older adults tend to live longer in their neighborhoods and surrounding areas due to physical, emotional, or economic needs, and are therefore more exposed to illness caused by neighborhood characteristics than younger adults^6^. In this context, the literature demonstrates the influence of neighborhood characteristics on the occurrence of depressive symptoms among older adults living in high-income countries^7,8^.

In this regard, studying Brazil becomes particularly relevant, as it is a middle-income country experiencing both population aging^9^ and rapid urbanization^10^. These processes have created unequal conditions in access to goods and services—such as residential and socio-spatial segregation—thus affecting how people live and occupy urban spaces^4^.

Given the above, this study investigated the association between depressive symptoms and neighborhood perception among older Brazilian adults living in urban areas, based on nationally representative data, and also examined differences between men and women.

## METHODS

### Study Design

This cross-sectional study used baseline data (2015–2016) from the *Estudo Longitudinal da Saúde dos Idosos Brasileiros* (ELSI-Brasil – Brazilian Longitudinal Study of Aging). ELSI-Brasil is a prospective, population-based cohort study designed to represent the non-institutionalized Brazilian population aged 50 years or older^11^. The baseline sample was selected via a multistage sampling design (municipality, census tract, and household) and included 9,412 participants residing in 70 municipalities across the five major geographic regions of Brazil. Data were collected via face-to-face interviews using a standardized multidimensional questionnaire^11^. Inclusion criteria for this study were: residence in an urban area and completion of the depressive symptoms questionnaire without the aid of a proxy respondent. Participants with missing or incomplete data on the outcome were excluded.

### Outcome

Depressive symptoms were assessed using the 8-item Center for Epidemiologic Studies –Depression Scale (CES-D-8)^12^, a scale commonly used in epidemiological studies with older adults^12^. It evaluates the presence of depressive symptoms based on the question: “How often you have felt this way during the past week…”, including the items: “you felt depressed,” “you felt everything you did was an effort,” “your sleep was restless,” “you were happy,” “you felt lonely,” “you enjoyed life,” “you felt sad,” and “you could not get going”^12^. Affirmative responses were summed, and scores ≥ 4 indicated the presence of depressive symptoms. This cutoff was adopted based on a Brazilian validation study^13^ that identified a cutoff point of 4 with sensitivity, specificity, and accuracy of 94.3%, 84.5%, and 90.9%, respectively^13^.

### Exposure Variables

Exposure variables consisted of seven indicators derived from 14 questions related to participants’ perceptions of the physical and social neighborhood environment, as shown in Box.

**Box:** Neighborhood perception variables. Brazilian Longitudinal Study of Aging (ELSI-Brasil), 2015–2016.

Indicator	Questions	Responses
**Physical environment**		
Urban mobility issues	When you leave your house, are you afraid of falling because of defective sidewalks?	Yes or No
	When you leave your house, are you concerned about the difficulty of getting on a bus, subway, or train?	
	When you leave your house, are you concerned about the difficulty of crossing the street?	
Noise pollution	In your neighborhood, does the noise from cars or buses bother you?	Yes or No
Physical disorder	In your neighborhood, are there houses or buildings with graffiti, broken windowpanes, damaged structures, or abandoned properties?	Yes or No
	In your neighborhood, is there garbage, debris, or overgrown grass on the streets, sidewalks, or vacant lots?	
	In the past three months, have you seen rats or signs of rats on your street?	
Lack of neighborhood pleasantness	Is the neighborhood where you live a good place to live?	Yes or No^a^
	Do the children or young people in your neighborhood treat adults with respect?	
	Is your neighborhood a good place for children to play and for raising adolescents?	
	Is it pleasant to walk, run, or ride a bicycle in your neighborhood?	
**Social environment**		
Violence	In the past 12 months, have you been a victim of theft, robbery, or home invasion?	Yes or No
Lack of social cohesion	Do you believe you can trust most people in your neighborhood?	Yes or No^a^
Feeling of insecurity	Thinking about crime and violence, which of the following statements best describes your neighborhood?	Yes or No^b^

^a^ Responses were categorized as “yes” and “no” (the options “more or less” and “no” were grouped).

^b^ Responses were categorized as “yes” (unsafe) and “no” (the options “very safe” and “safe” were grouped).

Composite indicators were created for questions related to physical disorder, urban mobility issues, and neighborhood pleasantness, and Cronbach’s alpha coefficients were estimated. For physical disorder and urban mobility issues, the condition was considered present when the participant responded positively to at least one of the three corresponding questions (Cronbach’s alpha = 0.42 and 0.70, respectively). For neighborhood pleasantness, absence was defined by a negative response to at least one of the four questions (Cronbach’s alpha = 0.63).

### Adjustment Variables

Adjustment variables were selected based on previous literature^1^ and included sociodemographic and health characteristics: age group (50–59, 60–69, 70–79, or ≥ 80 years); marital status (single, married/cohabiting, separated/divorced, or widowed); years of schooling (none, 1–4, 5–8, 9–11, or ≥ 12 years); self-reported race/skin color (White, Black, or Brown/Mixed-race), excluding Indigenous and Asian participants due to low frequency; *per capita* household income in tertiles (BRL) (1st tertile ≤ 620; 2nd tertile > 620–1,150; 3rd tertile > 1,150); living alone (yes/no); length of residence in the same municipality (years); self-rated health (very good/good, fair, or poor/very poor); and number of chronic diseases (0, 1–2, or ≥ 3), excluding depression and gestational hypertension and diabetes mellitus. The number of chronic diseases was determined by self-reported medical diagnosis of the following conditions: hypertension, diabetes mellitus, high cholesterol, heart attack, angina, heart failure, stroke, asthma, emphysema, chronic bronchitis or chronic obstructive pulmonary disease, arthritis or rheumatism, osteoporosis, chronic back issues (such as back, neck, or lower back pain, sciatica, vertebral or disc issues), cancer, chronic renal failure, Parkinson’s disease, and Alzheimer’s disease.

### Data Analysis

A descriptive analysis of categorical variables was conducted using relative frequencies and 95% confidence intervals (95%CI). The continuous variable was presented as mean and standard deviation (SD). Associations between exposure variables and the outcome were analyzed using prevalence ratios (PR) and 95%CIs, estimated via univariable and multivariable Poisson regression models. All analyses were stratified by gender, given the biological, psychological, and environmental differences between men and women regarding depression^1^.

In the multivariable analysis, each neighborhood perception variable was adjusted for sociodemographic and health variables, regardless of statistical significance. A variance inflation factor (VIF) analysis was performed for exposure and adjustment variables, revealing no multicollinearity. The significance level was set at 5%.

All analyses were conducted using Stata software (Stata Corporation, College Station, Texas), version 16.1, applying the survey (*svy*) command to account for the study’s complex sampling design and individual weights.

The ELSI-Brasil study was approved by the Research Ethics Committee of the Fundação Oswaldo Cruz – Minas Gerais (CAAE: 34649814.3.0000.5091). Participants provided written informed consent for all research procedures.

## RESULTS

Of the 9,412 participants in the cohort baseline, 7,935 (84.3%) lived in urban areas. Of these, 706 individuals (8.9%) were excluded for having completed the depressive symptoms module with the aid of a proxy respondent, and 114 (1.4%) were excluded due to missing outcome data.

Thus, 7,115 participants (4,041 women and 3,074 men) aged 50 years or older were included in this study. Among all participants, 50.1% were aged 50–59 years; 63.5% were married; 35.1% had 1–4 years of schooling; 45.4% self-identified as Brown/Mixed-race; 90.6% did not live alone; 48.0% had one or two chronic diseases; and 45.7% rated their health as very good or good. Regarding gender, 45.0% of women self-identified as White, whereas 46.5% of men self-identified as Brown/Mixed-race. The mean length of residence in the municipality was 41.0 (SD = 18.6) years for the total sample, 41.8 (SD = 19.3) years for women, and 40.2 (SD = 17.7) years for men (Table 1).

**Table 1:** Distribution of the sample and prevalence of depressive symptoms according to participants’ sociodemographic and health characteristics, for the total sample and by gender. Brazilian Longitudinal Study of Aging (ELSI-Brazil), 2015–2016.

Characteristic	Total		Women		Men	
	Distribution of the sample % (95%CI)	Prevalence % (95%CI)	Distribution of the sample % (95%CI)	Prevalence % (95%CI)	Distribution of the sample % (95%CI)	Prevalence % (95%CI)
Age group (years)						
50–59	50.1 (45.5–54.7)	36.7 (34.1–39.5)*	47.7 (44.2–51.3)	47.3 (44.2–50.4)*	52.8 (46.7–58.8)	25.7 (23.1–28.3)
60–69	30.1 (27.9–32.4)	33.6 (31.2–36.2)	30.8 (28.9–32.8)	42.3 (38.9–45.8)	29.3 (26.2–32.6)	23.0 (20.1–26.1)
70–79	14.6 (12.7–16.9)	30.9 (27.5–34.6)	15.7 (13.8–17.7)	37.1 (32.0–42.5)	13.4 (10.9–16.4)	22.5 (18.5–27.1)
≥ 80	5.2 (4.2–6.3)	35.9 (30.8–41.4)	5.8 (4.7–7.0)	41.8 (34.8–49.0)	4.5 (3.4–5.9)	27.2 (19.6–36.4)
Marital status						
Single	12.0 (10.5–13.6)	39.6 (35.4–43.9)*	14.0 (12.1–16.0)	43.5 (38.5–48.5)*	9.6 (8.1–11.3)	33.0 (26.4–40.4)*
Married	63.5 (60.7–66.4)	30.6 (28.7–32.6)	53.4 (50.5–56.3)	42.2 (39.4–45.1)	75.5 (72.5–78.2)	21.1 (19.3–22.9)
Divorced	11.2 (10.3–12.1)	44.6 (40.6–48.8)	12.5 (11.4–13.7)	51.5 (47.0–55.9)	9.6 (8.3–11.0)	34.2 (27.5–41.5)
Widowed	13.3 (11.6–15.2)	43.2 (39.4–47.0)	20.1 (18.1–22.3)	43.7 (39.5–47.9)	5.3 (4.3–6.7)	41.0 (34.7–47.6)
Years of schooling^a^						
None	9.2 (7.5–11.3)	46.2 (41.5–50.9)*	10.4 (8.6–12.6)	53.3 (48.0–58.5)*	7.8 (6.0–10.2)	35.1 (28.3–42.5)*
1 to 4	35.1 (32.7–37.6)	39.8 (37.2–42.4)	35.4 (32.6–38.3)	49.0 (46.2–51.7)	34.8 (32.0–37.6)	28.9 (25.8–32.0)
5 to 8	23.6 (22.1–25.3)	37.2 (34.0–40.5)	22.7 (20.6–25.0)	46.8 (41.8–51.8)	24.6 (23.2–26.4)	26.8 (23.8–30.0)
9 to 11	22.3 (20.6–24.1)	27.4 (24.3–30.6)	21.2 (19.3–23.2)	36.5 (32.1–41.1)	23.6 (21.2–26.0)	17.8 (14.8–21.2)
≥ 12	9.8 (8.3–11.4)	18.7 (15.0–23.0)	10.3 (8.7–12.8)	24.8 (20.1–30.0)	9.2 (7.4–11.4)	10.8 (6.8–16.7)
Self-reported race/skin color^b^						
White	44.8 (39.8–50.0)	31.2 (28.7–33.8)*	45.0 (39.3–50.7)	39.5 (36.2–42.9)*	44.7 (39.3–50.2)	21.5 (19.1–24.0)*
Black	9.8 (8.1–11.7)	39.9 (35.8–44.1)	10.6 (8.5–13.4)	48.1 (42.6–53.5)	8.8 (7.4–10.4)	28.3 (22.4–35.0)
Brown/Mixed-race	45.4 (41.2–49.6)	36.7 (34.5–39.0)	44.4 (39.9–48.9)	46.7 (43.6–49.6)	46.5 (41.8–51.3)	25.7 (23.2–28.4)
Per capita household income in tertiles (R$)						
1st tertile: ≤ 620	33.4 (29.8–37.2)	43.8 (41.5–46.0)*	36.0 (32.2–39.9)	51.0 (48.5–53.5)*	30.4 (26.6–34.5)	33.7 (30.1–37.6)*
2nd tertile: > 620 to 1,150	33.4 (31.7–35.2)	36.2 (33.9–38.7)	33.5 (31.3–35.7)	46.2 (42.3–50.1)	33.3 (31.4–35.3)	24.6 (21.8–27.6)
3rd tertile: > 1,150	33.2 (30.1–36.4)	24.7 (22.6–27.0)	30.5 (27.4–33.9)	32.8 (29.9–35.9)	36.3 (32.5–40.1)	16.7 (14.5–19.1)
Living alone						
Yes	9.4 (8.3–10.6)	40.1 (36.5–43.8)*	11.3 (10.0–12.8)	42.0 (37.2–46.8)	7.1 (5.9–8.5)	36.6 (31.3–42.3)*
No	90.6 (89.4–91.7)	34.4 (32.6–36.2)	88.7 (87.1–90.0)	44.1 (41.9–46.2)	92.9 (91.4–94.1)	23.6 (21.8–25.4)
Length of residence (years)^c^ [mean (SD)]	41.0 (18.6)	41.3 (18.7)	41.8 (19.3)	41.8 (18.8)	40.2 (17.7)	40.1 (18.2)
Number of chronic diseases^d^						
0	16.4 (15.1–17.8)	21.0 (17.8–24.5)*	10.9 (9.6–12.5)	25.8 (20.9–31.4)*	22.7 (21.0–24.5)	18.3 (14.7–22.5)*
1 to 2	48.0 (46.0–50.1)	29.5 (27.3–31.9)	44.7 (42.2–47.1)	37.1 (34.3–40.0)	51.9 (49.4–54.4)	22.0 (19.3–24.9)
≥ 3	35.6 (33.1–38.1)	47.2 (44.2–50.2)	44.4 (42.0–46.8)	54.2 (51.0–57.2)	25.4 (22.6–28.2)	33.0 (29.2–37.0)
Self-rated health^e^						
Very good or good	45.7 (42.7–48.7)	21.5 (19.5–23.7)*	45.1 (42.0–48.1)	29.1 (26.1–32.3)*	46.4 (42.5–50.3)	13.0 (10.9–15.3)*
Fair	43.3 (41.2–45.4)	40.2 (38.4–42.4)	42.9 (40.6–45.3)	50.1 (47.1–53.1)	43.6 (40.9–46.3)	28.8 (25.9–31.9)
Poor or very poor	11.0 (9.8–12.5)	69.6 (66.2–72.7)	12.0 (10.7–13.5)	76.9 (72.3–80.9)	10.0 (8.2–12.0)	59.3 (54.3–64.1)
n (unweighted)	7,115		4,041		3,074	

95%CI: 95% confidence interval; SD: standard deviation; W: women; M: men.

Missing data

^a^ 39 (0.6%), W = 25 and M = 14.

^b^ 456 (6.4%), W = 262 and M = 194, excluding Asian (90; 0.99%) and Indigenous (220; 2.43%).

^c^ 71 (1%), W = 61 and M = 10.

^d^ 267 (3.8%), W = 159 and M = 108.

^e^ 16 (0.2%), W = 12 and M = 4.

* p < 0.05.

The prevalence of depressive symptoms in the total sample was 34.9% (95%CI: 33.2–36.7), 43.8% (95%CI: 41.5–45.9) among women, and 24.5% (95%CI: 22.8–26.3) among men. In the univariate analysis, for the total sample and by gender, depressive symptoms were associated with sociodemographic and health characteristics (Table 1), as well as the participants’ perception of the neighborhood (Table 2).

**Table 2:** Description of the sample and prevalence of depressive symptoms according to perceived neighborhood characteristics, for the total sample and by gender. Brazilian Longitudinal Study of Aging (ELSI-Brazil), 2015–2016.

Neighborhood characteristics	Total		Women		Men	
	Sample description % (95%CI)	Prevalence % (95%CI)	Sample description % (95%CI)	Prevalence % (95%CI)	Sample description % (95%CI)	Prevalence % (95%CI)
Urban mobility issues^a^						
No	34.7 (32.0–37.4)	22.9 (20.4–25.4)*	25.7 (23.2–28.3)	30.3 (26.3–34.5)*	45.0 (41.4–48.6)	18.0 (15.4–20.8)*
Yes	65.3 (62.6–67.9)	41.5 (39.3–43.8)	74.3 (71.7–76.8)	48.9 (46.5–51.3)	55.0 (51.3–58.5)	30.0 (27.2–32.9)
Noise pollution^b^						
No	70.0 (68.0–71.9)	31.9 (29.9–33.9)*	70.1 (67.8–72.4)	41.2 (38.6–43.8)*	69.8 (67.5–72.0)	21.0 (18.7–23.4)*
Yes	30.0 (28.1–32.0)	41.9 (39.2–44.6)	29.9 (27.6–32.2)	50.1 (46.9–53.2)	30.2 (28.0–32.5)	32.5 (29.4–35.8)
Physical disorder^c^						
No	36.6 (33.1–40.2)	28.9 (26.5–31.3)*	36.0 (32.8–39.3)	38.9 (35.4–42.4)*	37.3 (32.9–41.8)	17.8 (15.3–20.6)*
Yes	63.4 (59.8–66.8)	38.2 (36.2–40.2)	64.0 (60.7–67.1)	46.7 (44.4–49.0)	62.7 (58.1–67.1)	28.2 (26.3–30.5)
Neighborhood pleasantness^d^						
No	7.6 (6.5–8.8)	48.7 (44.1–53.2)*	7.1 (6.0–8.4)	60.4 (54.2–66.3)*	8.1 (6.6–9.9)	37.1 (31.3–43.2)*
Yes	92.4 (91.2–93.5)	33.7 (31.9–35.5)	92.9 (91.5–94.0)	42.7 (40.4–44.9)	91.9 (90.1–93.4)	23.6 (21.8–25.5)
Violence^e^						
No	92.8 (91.9–93.5)	34.2 (32.4–36.1)*	93.1 (92.1–94.0)	43.2 (41.0–45.4)*	92.3 (91.1–93.4)	23.7 (21.9–25.6)*
Yes	7.2 (6.5–8.1)	43.5 (38.8–48.2)	6.9 (6.0–7.9)	53.0 (46.6–59.1)	7.7 (6.6–8.9)	33.6 (27.3–40.4)
Social cohesion^f^						
No	46.5 (43.8–49.1)	41.6 (39.6–43.5)*	47.8 (44.2–51.4)	51.2 (48.7–53.6)*	44.9 (42.2–47.6)	29.7 (26.9–32.7)*
Yes	53.5 (50.9–56.2)	29.1 (26.9–31.3)	52.2 (48.6–55.8)	37.2 (34.1–40.2)	55.1 (52.4–57.8)	20.2 (18.0–22.5)
Perceived insecurity^g^						
No	61.1 (57.7–64.3)	38.3 (35.6–41.1)*	61.2 (58.0–64.3)	48.0 (44.7–51.4)*	60.9 (56.5–65.2)	27.3 (23.9–30.8)*
Yes	38.9 (35.6–42.3)	32.5 (30.6–34.3)	38.8 (35.6–42.0)	41.1 (38.4–43.9)	39.1 (34.8–43.5)	22.5 (20.2–24.8)
n (unweighted)	7,115		4,041		3,074	

95%CI: 95% confidence interval; W: women; M: men.

Missing data

^a^ 315 (4.4%); W = 202 and M = 113.

^b^ 230 (3.2%); W = 144 and M = 86.

^c^ 356 (5.0%); W = 235 and M = 121.

^d^ 401 (5.6%); W = 299 and M = 102.

^e^ 228 (3.2%); W = 143 and M = 85.

^f^ 67 (0.9%); W = 55 and M = 12.

^g^ 323 (4.5%); W = 211 and M = 112.

* p < 0.05.

For the total sample, 65.3% reported issues with urban mobility, 30.0% reported noise pollution, 63.4% reported physical disorder, 7.6% reported absence of pleasantness in the neighborhood, 7.2% reported violence, 46.5% reported lack of social cohesion, and 38.9% reported a sense of insecurity (Table 2).

In the multivariable analysis, significant associations were observed for the total sample (T), women (W), and men (M) and prevalence ratios (PR) between depressive symptoms and the following variables: urban mobility issues (T: PR = 1.39; 95%CI: 1.25–1.56; W: PR = 1.37; 95%CI: 1.21–1.56; M: PR = 1.38; 95%CI: 1.15–1.64); noise pollution (T: PR = 1.27; 95%CI: 1.19–1.35; W: PR = 1.18; 95%CI: 1.10–1.27; M: PR = 1.46; 95%CI: 1.26–1.70); physical disorder (T: PR = 1.22; 95%CI: 1.12–1.33; W: PR = 1.15; 95%CI: 1.05–1.25; M: PR = 1.43; 95%CI: 1.22–1.68); lack of pleasantness on the neighborhood (T: PR = 1.24; 95%CI: 1.12–1.39; W: PR = 1.25; 95%CI: 1.08–1.43; M: PR = 1.22; 95%CI: 1.04–1.43); violence (T: PR = 1.31; 95%CI: 1.17–1.45; W: PR = 1.25; 95%CI: 1.10–1.41; M: PR = 1.44; 95%CI: 1.17–1.77); and lack of social cohesion (T: PR = 1.26; 95%CI: 1.17–1.36; W: PR = 1.27; 95%CI: 1.16–1.38; M: PR = 1.26; 95%CI 1.10–1.46). A gender difference was observed only for sense of insecurity, which showed a significant association for the total sample (PR = 1.13; 95%CI: 1.05–1.21) and among women (PR = 1.12; 95%CI: 1.03–1.21), but not among men (Table 3).

**Table 3:** Univariate and multivariate analyses of the associations between neighborhood perception and depressive symptoms, for the total sample and by gender. Brazilian Longitudinal Study of Aging (ELSI-Brasil), 2015–2016.

Characteristic	Total		Women		Men	
	Crude PR (95%CI)	Adjusted PR (95%CI)^a^	Crude PR (95%CI)	Adjusted PR (95%CI)^a^	Crude PR (95%CI)	Adjusted PR (95%CI)^a^
Urban mobility issues	1.82 (1.63–2.03)	1.39 (1.25–1.56)	1.61 (1.41–1.85)	1.37 (1.21–1.56)	1.67 (1.38–2.01)	1.38 (1.15–1.64)
Noise pollution	1.31 (1.22–1.41)	1.27 (1.19–1.35)	1.21 (1.12–1.31)	1.18 (1.10–1.27)	1.55 (1.32–1.81)	1.46 (1.26–1.70)
Physical disorder	1.32 (1.23–1.43)	1.22 (1.12–1.33)	1.20 (1.10–1.32)	1.15 (1.05–1.25)	1.59 (1.35–1.87)	1.43 (1.22–1.68)
Lack of neighborhood pleasantness	1.44 (1.31–1.59)	1.24 (1.22–1.39)	1.42 (1.27–1.59)	1.25 (1.08–1.43)	1.57 (1.32–1.86)	1.22 (1.04–1.43)
Violence	1.27 (1.14–1.41)	1.31 (1.17–1.45)	1.23 (1.09–1.38)	1.25 (1.10–1.41)	1.41 (1.14–1.75)	1.44 (1.17–1.77)
Lack of social cohesion	1.43 (1.32–1.54)	1.26 (1.17–1.36)	1.38 (1.26–1.51)	1.27 (1.16–1.38)	1.47 (1.26–1.71)	1.26 (1.10–1.46)
Feeling of insecurity	1.18 (1.09–1.27)	1.13 (1.05–1.21)	1.17 (1.06–1.28)	1.12 (1.03–1.21)	1.21 (1.02–1.44)	1.15 (0.98–1.35)^b^

PR: prevalence ratio; 95%CI: 95% confidence interval.

^a^ Models adjusted for sociodemographic and health variables: age group, marital status, years of schooling, self-reported race/skin color, per capita household income in tertiles, living alone, length of residence (in years), number of chronic diseases (excluding depression and gestecional hypertension and diabetes mellitus), and self-rated health.

^b^ p ≥ 0.05 (not significant).

## DISCUSSION

Older adults living in urban areas in Brazil who presented depressive symptoms reported a significantly higher perception of urban mobility issues, noise pollution, physical disorder, violence, lack of neighborhood pleasantness, and social cohesion in the total sample and in both strata. The report of feeling unsafe among those with depressive symptoms was significantly higher in the total sample and among women only.

Our findings for the total sample are consistent with previous literature. Neighborhood mobility depends on well-designed streets, convenient access to transportation, and a pleasant physical environment^14^. Our results show a 39% increase in the prevalence of depressive symptoms among participants who reported perceiving urban mobility issues, such as fear of falling due to defective sidewalks, concern about the difficulty of getting on a bus, subway, or train, or difficulty crossing the street. A study conducted in a municipality in southern Brazil^15^ showed that the presence of crosswalks, traffic lights, or pedestrian bridges contributed to a reduced likelihood of depressive symptoms among older adults (≥ 60 years). Similarly, a study of older Chinese adults (≥ 60 years) on walkability^16^—classified based on the proportion of visible sky on the street—showed an increase in depression scores associated with reduced neighborhood walkability.

Regarding noise pollution, the results showed that the perception of road traffic noise was associated with a 27% increase in the prevalence of depressive symptoms. These findings are consistent with Yun et al.^17^, Lin et al.^18^, and Orban et al.^19^. In the study by Yun et al.^17^, the authors showed that people of different ages, including older adults (≥ 60 years), living in areas with high exposure to environmental noise were more likely to develop mild to moderate depressive symptoms compared to those with lower exposure. Lin et al.^18^, in a community-based cross-sectional study, identified an association between 24-hour exposure to road traffic noise and a higher prevalence of depression among residents aged 30 to 70 years. Orban et al.^19^, in a five-year prospective cohort study conducted in Germany with participants aged 45 to 75 years, observed that residential road traffic noise increased the risk of elevated depressive symptoms among middle-aged and older adults.

Physical disorder and pleasantness are interrelated indicators whose components include the presence of vacant buildings or houses; lack of pleasant spaces for children, youth, and adolescents; litter on streets and sidewalks; vacant lots with garbage or overgrown grass; broken windows; and deteriorated walls^20^. Our results show that the presence of physical disorder and lack of pleasantness increased the prevalence of depressive symptoms by 22% and 24%, respectively. These findings are consistent with those of Pai and Kim^21^ (population aged 40 to 96 years) and Wang et al.^22^ (older adults ≥ 60 years), who found a positive association between physical disorder and depressive symptoms, as well as with Helbich et al.^23^, who identified a negative correlation between perceived neighborhood pleasantness and depression severity among Dutch adults aged 18 to 65 years.

Social cohesion is a psychosocial resource that protects against stress and depression, as it provides affective support among neighbors^24^. Our results identified a 26% increase in the prevalence of depressive symptoms among participants who perceived a lack of social cohesion. This result converges with that of Helbich et al.^23^, who found an inverse correlation between depression severity and perceived social cohesion in a cross-sectional study with Dutch adults (18 to 65 years), and with Ruiz et al.^25^, who demonstrated a higher density of incident depressive symptoms in the lowest tertiles of perceived social cohesion over 12 years among English adults (≥ 50 years).

The construct of violence refers to having been a victim of physical or psychological violence, theft, robbery, or home invasion, and to the perception of crime^26,27^. It is related to insecurity, which corresponds both to the feeling of safety in the neighborhood and to perceptions of crime in the area^27^. Our results show a 31% increase in the prevalence of depressive symptoms among participants who reported having been victims of theft, robbery, or home invasion in the previous 12 months, and a 12% increase among women who reported feeling unsafe due to crime and violence. The observed increase in the prevalence of depressive symptoms is consistent with Ivey et al.^26^, who found that older adults (≥ 65 years) in the U.S., who reported lower safety from crime, were more likely to be depressed, as well as with Cândido et al.^15^, who found a lower likelihood of depressive symptoms among Brazilian older adults (≥ 60 years) who felt safe walking during the day and at night in the municipality of Balneário Arroio do Silva, Santa Catarina. Another study with older adults (50 to 74 years) residing in New Jersey, which assessed the correlation between violence rates and perceived safety, found that older adults living in neighborhoods with higher rates of violent crime and greater concern about safety showed higher levels of depressive symptoms^27^.

The perception of insecurity among women may reflect a heightened perception of vulnerability to violence in Brazil^9^. In the Brazilian social and cultural context, violence against women has become an increasing phenomenon involving both lethal and non-lethal assaults, occurring in domestic, community, and institutional settings, with men being the main perpetrators^9^. Moreover, in the case of older women, gender intersects with the biological, economic, and social changes associated with aging, which may contribute to increased dependence in daily activities and, consequently, greater vulnerability and predisposition to violence perpetrated by both men and women in caregiving roles^9^. Our finding is consistent with Campos^28^, who found that older Brazilian women (≥ 60 years) from a municipality in southeastern Brazil were more likely to feel unsafe in the face of urban violence, precisely because women are the main victims^28^.

Although depression is a multifactorial phenomenon, exposure to stressful situations may lie in its causal pathway^29^. Custrona et al.^5^ argue that stress plays an important explanatory role in the association between neighborhood characteristics and the development of depression. The authors propose an explanatory model that considers the neighborhood as a producer of daily stress levels, arising from increased vulnerability to negative events and the loss of social ties, which lead to depression. Stress responses can wear down the body and produce pathological changes, such as hippocampal neuron loss, reduced neurogenesis, and cognitive issues resulting from excessive cortisol production^29^.

In this sense, living in an environment with physical disorder can generate feelings of helplessness and stress among residents^5^. Likewise, noise pollution can contribute to daily doses of stress, exacerbating the environmental impacts on psychological well-being. In addition, older adults with urban mobility issues who live in neighborhoods with environmental barriers experience reduced opportunities for social interaction, contributing to isolation and the emergence of depressive symptoms^30^. Finally, violence and insecurity act as environmental stressors^16,27^, generating a constant sense of danger and further contributing to social isolation.

Moreover, social cohesion buffers stress arising from negative neighborhood perceptions and its impact on residents’ mental health, serving as a potential protective resource against the development of depression by reducing isolation and increasing social interaction^24^. Therefore, the absence of this attribute contributes to increased neighborhood stress and its effects on the prevalence of depressive symptoms.

This study has some limitations that should be mentioned. First, the self-reported nature of the scale used to measure the outcome may be subject to respondent bias. Participants may, for example, underreport depressive symptoms due to lack of awareness or cultural factors. Moreover, the cross-sectional design of the study requires caution in interpreting the results due to possible reverse causality bias. Nevertheless, our findings were consistent with previous cross-sectional and longitudinal studies^15^. Notably, this study has several strengths, including a robust, nationally representative sample of Brazilian adults aged 50 years or older in a middle-income country.

By investigating different domains of neighborhood perception (physical and social environment), the results reveal a close relationship between depressive symptoms in older Brazilian adults and urban space. Our findings indicate that the presence of urban mobility issues, noise pollution, physical disorder, violence, lack of neighborhood pleasantness, and social cohesion increased the prevalence of depressive symptoms regardless of gender. The feeling of insecurity was associated with a higher prevalence of depressive symptoms only among women, which may be related to the Brazilian context of gender-based violence and its implications for the production of specific vulnerabilities in women’s aging processes.

The results highlight the importance of intersectoral action among health, urban mobility, public safety, and city planning policies, focusing on promoting the mental health of all residents, especially older women and men living in urban areas. Therefore, reducing older adults’ negative perceptions of their neighborhoods may improve well-being and mental health in later life, contributing to a lower prevalence of depressive symptoms.

## Data Availability

The research data are available upon registration and request through the following link: https://elsi.cpqrr.fiocruz.br/data-access/
