# Supplementary material for: Neighborhood perception and depressive symptoms: the ELSI-Brasil national study, 2015–2016
Source: Rev Saude Publica. 2025 Dec 8;59:e42. doi: 10.11606/s1518-8787.2025059006948 (PMC12674688; doi:10.11606/s1518-8787.2025059006948)
Supplement: Translation

# Translation: Percepção da vizinhança e sintomas depressivos: estudo nacional ELSI-Brasil, 2015–2016

**Authors:** Pablo Cardozo Roccon, Amanda Cristina de Souza Andrade, Débora Moraes Coelho, Bruno de Souza Moreira, Luciana de Souza Braga, Maria Fernanda Lima-Costa, Waleska Teixeira Caiaffa

**Affiliations:** I Universidade Federal de Minas Gerais. Faculdade de Medicina. Departamento de Medicina Preventiva e Social. Belo Horizonte, MG, Brasil; II Universidade Federal de Minas Gerais. Observatório de Saúde Urbana de Belo Horizonte. Belo Horizonte, MG, Brasil; III Universidade Federal de Mato Grosso. Instituto de Saúde Coletiva. Programa de Pós-Graduação em Saúde Coletiva. Cuiabá, MT, Brasil; IV Fundação Oswaldo Cruz. Instituto René Rachou. Belo Horizonte, MG, Brasil; V Núcleo de Estudos em Saúde Pública e Envelhecimento. Belo Horizonte, MG, Brasil; VI Universidade Federal de Minas Gerais. Faculdade de Medicina. Programa de Pós-Graduação em Saúde Pública. Belo Horizonte, MG, Brasil

**Keywords:** Sintomas Depressivos, Ambiente da Vizinhança, Efeitos da Vizinhança, Percepção, Pessoa de Meia-Idade, Idoso

## Abstract

**OBJETIVO:**

Investigar a associação entre sintomas depressivos e a percepção da vizinhança entre adultos mais velhos residentes em áreas urbanas.

**MÉTODOS:**

Trata-se de um estudo transversal incluindo 7.115 indivíduos (≥ 50 anos) da linha de base (2015–2016) do Estudo Longitudinal da Saúde dos Idosos Brasileiros (ELSI-Brasil), residentes em áreas urbanas, com informações completas sobre o desfecho e respostas sem auxílio de respondente substituto. O desfecho foi medido pela *8-item Center for Epidemiologic Studies–Depression Scale* (CES-D-8) (ponto de corte ≥ 4) e a exposição pela percepção da vizinhança sobre a área de residência dos participantes. Foi realizada regressão de Poisson univariada e múltipla, com ajuste de cada variável de exposição pelas características sociodemográficas e condições de saúde.

**RESULTADOS:**

A prevalência de sintomas depressivos foi de 34,9%, sendo 43,8% e 24,5% entre mulheres e homens, respectivamente. Na análise múltipla, problemas de mobilidade urbana, presença de poluição sonora, desordem física, violência, ausência de agradabilidade do bairro, coesão social e sensação de insegurança foram significativamente associados ao aumento na prevalência de sintomas depressivos para o total da amostra e entre as mulheres. Já entre os homens, exceto pela sensação de insegurança, as demais variáveis também foram associadas significativamente ao aumento na prevalência de sintomas depressivos.

**CONCLUSÃO:**

Os resultados apontam para a importância de uma ação intersetorial entre as políticas de saúde, mobilidade urbana, segurança pública e planejamento e organização das cidades com foco na promoção à saúde mental de adultos mais velhos residentes em áreas urbanas.

## INTRODUÇÃO

Os sintomas depressivos podem ter importante impacto na saúde, na qualidade de vida e no bem-estar psicossocial dos adultos mais velhos^1^. Segundo estudos de metanálise, no mundo, 35,1% dos adultos mais velhos apresentam sintomas depressivos^2^, enquanto, no Brasil, a prevalência foi de 21,0%^3^.

Estudos têm apontado que o ambiente urbano (natural, construído, social e econômico) pode favorecer a saúde ou a produção de iniquidades de seus residentes^4^. Características do ambiente social e físico da vizinhança podem suscitar doses diárias de estresse, elevando a prevalência de sintomas depressivos em adultos mais velhos^5^.

Pessoas idosas tendem a viver mais tempo nas vizinhanças e proximidades de sua residência em razão de necessidades físicas, emocionais ou econômicas, estando mais expostas a chances de adoecimento decorrentes das características da vizinhança se comparadas a adultos mais jovens^6^. Diante desse cenário, a literatura demonstra a influência das características da vizinhança na ocorrência de sintomas depressivos entre adultos mais velhos residentes de países de alta renda^7,8^.

Nessa direção, estudar o Brasil ganha relevância por se tratar de um país de média renda convivendo com o crescimento de sua população idosa^9^ e acelerado processo de urbanização^10^, que tem produzido condições desiguais de acesso a bens e serviços, como segregação econômica residencial e socioespacial, e, assim, impacta o modo de viver e ocupar os espaços urbanos^4^.

Diante do exposto, o presente estudo investigou a associação entre sintomas depressivos e a percepção da vizinhança entre adultos mais velhos brasileiros residentes em áreas urbanas baseado em dados de uma amostra nacionalmente representativa, considerando também a estratificação entre homens e mulheres.

## MÉTODOS

### Desenho do Estudo

Trata-se de um estudo transversal com dados da linha de base (2015–2016) do Estudo Longitudinal da Saúde dos Idosos Brasileiros (ELSI-Brasil). O ELSI-Brasil é uma coorte prospectiva, de base domiciliar, delineada para representar a população brasileira não institucionalizada com 50 anos ou mais^11^. A amostra da linha de base foi delineada a partir de diferentes estágios de seleção (município, setor censitário e domicílio), composta por 9.412 participantes, residentes em 70 municípios, distribuídos nas cinco macrorregiões geográficas brasileiras. Os dados foram obtidos a partir de entrevistas face a face utilizando um questionário multidimensional padronizado^11^. Os critérios de inclusão no presente estudo foram: residir em áreas urbanas e ter respondido ao questionário de sintomas depressivos sem o auxílio de respondente substituto. Participantes com dados faltantes ou incompletos em relação ao desfecho foram excluídos.

### Desfecho

Os sintomas depressivos foram avaliados pela *8-item Center for Epidemiologic Studies –Depression Scale* (CES-D-8)^12^. Escala comumente utilizada em estudos epidemiológicos com adultos mais velhos^12^ que avalia a presença de sintomas depressivos a partir do questionamento sobre oito itens relacionados a última semana: “você se sentiu deprimido?”, “você sentiu que as coisas estavam mais difíceis do que costumavam ser antes?”, “você sentiu que o seu sono não era reparador?”, “você se sentiu feliz?”, “você se sentiu solitário?”, “você aproveitou ou sentiu prazer pela vida?”, “você se sentiu triste?” e “você sentiu que não conseguiria levar adiante as suas coisas?”^12^. As respostas afirmativas aos itens da CES-D-8 foram somadas e escores ≥ 4 indicaram a presença de sintomas depressivos. Esse ponto de corte foi adotado por um estudo brasileiro^13^ após análise de validação para o Brasil, que identificou ponto de corte de 4 com sensibilidade, especificidade e acurácia de 94,3%, 84,5%, 90,9%, respectivamente^13^.

### Variáveis de Exposição

As variáveis de exposição consistiram em sete indicadores produzidos a partir de 14 questões relacionadas à percepção do ambiente físico e social da vizinhança apresentadas no Quadro.

**Quadro:** Variáveis de percepção da vizinhança. Estudo Longitudinal da Saúde dos Idosos Brasileiros (ELSI-Brasil), 2015–2016.

Indicador	Questões	Respostas
**Ambiente Físico**		
Problemas de mobilidade urbana	Quando vai sair de casa, o(a) senhor(a) tem medo de cair por causa de defeitos nos passeios?	Sim ou não
	Quando vai sair de casa, o(a) senhor(a) tem preocupação com a dificuldade para subir no ônibus, metrô ou trem?	
	Quando vai sair de casa, o(a) senhor(a) tem preocupação com a dificuldade para atravessar a rua?	
Poluição sonora	Na sua vizinhança, o ruído/barulho de carros ou ônibus o(a) incomoda?	Sim ou não
Desordem física	Na sua vizinhança, existem casas ou prédios com pichação, vidros das janelas quebrados, esburacados ou abandonados?	Sim ou não
	Na sua vizinhança, existe lixo, entulho, mato alto nas ruas, no passeio público ou nos lotes vagos?	
	Nos últimos três meses, o(a) senhor(a) viu ratos ou sinais de ratos na rua onde mora?	
Ausência de agradabilidade do bairro	O bairro onde o(a) senhor(a) mora é um bom lugar para se viver?	Sim ou não^a^
	As crianças ou os jovens da sua vizinhança tratam os adultos com respeito?	
	A sua vizinhança é um bom lugar para as crianças brincarem e para criar adolescentes?	
	É agradável fazer caminhada, correr ou andar de bicicleta na sua vizinhança?	
**Ambiente social**		
Violência	Nos últimos 12 meses, o(a) senhor(a) foi vítima de furto, roubo ou teve a sua residência invadida?	Sim ou não
Ausência de coesão social	O(A) senhor(a) acredita que pode confiar na maioria das pessoas na vizinhança?	Sim ou não^a^
Sensação de insegurança	Pensando em crimes e violência, qual das frases que vou ler a seguir define melhor a sua vizinhança?	Sim ou não^b^

^a^ As respostas foram categorizadas em sim e não (agrupou-se “mais ou menos” e “não”).

^b^ As respostas foram categorizadas em sim (insegura) e não (agrupou-se “muito segura” e “segura”).

Foram criados indicadores compostos para as perguntas relativas às percepções de desordem física, problemas de mobilidade urbana e agradabilidade do bairro, com cálculo do coeficiente alfa de Cronbach. Para os indicadores de *Desordem física e Problemas de mobilidade urbana,* considerou-se a presença quando o participante respondeu positivamente a pelo menos uma das três questões correspondentes (alfa de Cronbach = 0,42 e 0,70, respectivamente). Para o indicador de *Agradabilidade do bairro,* a ausência foi definida pela resposta negativa a uma das quatro questões (alfa de Cronbach = 0,63).

### Variáveis de Ajuste

As variáveis de ajuste foram selecionadas com base em literatura prévia^1^ e incluíram características sociodemográficas e de saúde: faixa etária (50–59, 60–69, 70–79 ou ≥ 80 anos); estado civil (solteiro, casado/amasiado/união estável, separado/divorciado ou viúvo); anos de estudo (nunca estudou, 1 a 4, 5 a 8, 9 a 11 ou ≥ 12 anos); cor autorreferida (branco, preto ou pardo), com exclusão de indígenas ou amarelos por baixa frequência; renda familiar *per capita* em tercis (R$) (1º tercil: ≤ 620, 2º tercil: > 620 a 1.150 ou 3º tercil: > 1.150); residir sozinho (sim ou não); tempo de residência no mesmo município (em anos); autoavaliação de saúde (muito boa/boa, regular ou ruim/muito ruim); e número de doenças crônicas (0, 1 a 2 ou ≥ 3), excluindo depressão, hipertensão arterial ou diabetes mellitus na gravidez. O número de doenças crônicas foi definido pelo autorrelato de diagnóstico médico das seguintes doenças: hipertensão arterial, diabetes mellitus, colesterol alto, infarto do coração, angina do peito, insuficiência cardíaca, acidente vascular cerebral, asma, enfisema, bronquite crônica ou doença pulmonar obstrutiva crônica, artrite ou reumatismo, osteoporose, problema crônico de coluna (como dor nas costas, no pescoço, lombalgia, dor ciática, problemas nas vértebras ou disco), câncer, insuficiência renal crônica, doença de Parkinson e doença de Alzheimer.

### Análise dos Dados

Realizou-se uma análise descritiva das variáveis categóricas, utilizando frequências relativas e respectivos intervalos de confiança de 95% (IC95%). A variável contínua foi apresentada como média e desvio padrão (DP). As associações entre as variáveis de exposição e o desfecho foram analisadas por meio de razões de prevalência (RP) e IC95%, estimadas pela regressão de Poisson univariada e múltipla. Todas as análises foram estratificadas por sexo, considerando as diferenças entre homens e mulheres quanto a fatores biológicos, psicológicos e ambientais relacionados à depressão^1^.

Na análise múltipla, cada variável de percepção da vizinhança foi ajustada pelas variáveis sociodemográficas e de saúde, independentemente de sua significância estatística. Foi realizada uma análise de fator de inflação de variância (VIF) para as variáveis de exposição e ajuste, na qual não foi encontrada multicolinearidade. O nível de significância adotado foi de 5%.

Todas as análises foram feitas com o *software* Stata (Stata Corporation, College Station, Texas), versão 16.1, utilizando o comando *survey* (*svy)* para considerar o desenho amostral complexo da pesquisa e o peso dos indivíduos.

O ELSI-Brasil foi aprovado pelo Comitê de Ética da Fundação Oswaldo Cruz, Minas Gerais (CAAE: 34649814.3.0000.5091). Os participantes assinaram termos de consentimento livre e esclarecido para cada procedimento da pesquisa.

## RESULTADOS

Entre os 9.412 participantes da linha de base da coorte, 7.935 (84,3%) residiam em áreas urbanas. Destes, foram excluídos 706 (8,9%) indivíduos por apresentarem respostas do bloco de sintomas depressivos com auxílio de respondente substituto e 114 (1,4%) indivíduos com ausência de informação do desfecho.

Assim, 7.115 participantes (4.041 mulheres e 3.074 homens) com 50 anos ou mais participaram deste estudo. Do total de participantes, 50,1% tinham entre 50 e 59 anos; 63,5% eram casados; 35,1% possuíam de 1 a 4 anos de estudo; 45,4% autodeclararam-se pardos; 90,6% não residiam sozinhos; 48,0% apresentavam 1 ou 2 doenças crônicas; e 45,7% avaliavam sua saúde como muito boa ou boa. Em relação ao sexo, 45,0% das mulheres se autodeclararam brancas e 46,5% dos homens como pardos. O tempo médio de residência no município foi de 41,0 (18,6) anos para o total da amostra, sendo 41,8 (19,3) anos para mulheres e 40,2 (17,7) anos para homens (Tabela 1).

**Tabela 1:** Distribuição da amostra e prevalência de sintomas depressivos segundo as características sociodemográficas e de saúde dos participantes do estudo, para o total da amostra e por sexo. Estudo Longitudinal da Saúde dos Idosos Brasileiros (ELSI-Brasil), 2015–2016.

Variável	Total		Feminino		Masculino	
	Distribuição da amostra % (IC95%)	Prevalência % (IC95%)	Distribuição da amostra % (IC95%)	Prevalência % (IC95%)	Distribuição da amostra % (IC95%)	Prevalência % (IC95%)
Faixa etária (anos)						
50–59	50,1 (45,5–54,7)	36,7 (34,1–39,5)*	47,7 (44,2–51,3)	47,3 (44,2–50,4)*	52,8 (46,7–58,8)	25,7 (23,1–28,3)
60–69	30,1 (27,9–32,4)	33,6 (31,2–36,2)	30,8 (28,9–32,8)	42,3 (38,9–45,8)	29,3 (26,2–32,6)	23,0 (20,1–26,1)
70–79	14,6 (12,7–16,9)	30,9 (27,5–34,6)	15,7 (13,8–17,7)	37,1 (32,0–42,5)	13,4 (10,9–16,4)	22,5 (18,5–27,1)
≥ 80	5,2 (4,2–6,3)	35,9 (30,8–41,4)	5,8 (4,7–7,0)	41,8 (34,8–49,0)	4,5 (3,4–5,9)	27,2 (19,6–36,4)
Estado civil						
Solteiro	12,0 (10,5–13,6)	39,6 (35,4–43,9)*	14,0 (12,1–16,0)	43,5 (38,5–48,5)*	9,6 (8,1–11,3)	33,0 (26,4–40,4)*
Casado	63,5 (60,7–66,4)	30,6 (28,7–32,6)	53,4 (50,5–56,3)	42,2 (39,4–45,1)	75,5 (72,5–78,2)	21,1 (19,3–22,9)
Divorciado	11,2 (10,3–12,1)	44,6 (40,6–48,8)	12,5 (11,4–13,7)	51,5 (47,0–55,9)	9,6 (8,3–11,0)	34,2 (27,5–41,5)
Viúvo	13,3 (11,6–15,2)	43,2 (39,4–47,0)	20,1 (18,1–22,3)	43,7 (39,5–47,9)	5,3 (4,3–6,7)	41,0 (34,7–47,6)
Anos de estudo^a^						
Nunca estudou	9,2 (7,5–11,3)	46,2 (41,5–50,9)*	10,4 (8,6–12,6)	53,3 (48,0–58,5)*	7,8 (6,0–10,2)	35,1 (28,3–42,5)*
1 a 4	35,1 (32,7–37,6)	39,8 (37,2–42,4)	35,4 (32,6–38,3)	49,0 (46,2–51,7)	34,8 (32,0–37,6)	28,9 (25,8–32,0)
5 a 8	23,6 (22,1–25,3)	37,2 (34,0–40,5)	22,7 (20,6–25,0)	46,8 (41,8–51,8)	24,6 (23,2–26,4)	26,8 (23,8–30,0)
9 a 11	22,3 (20,6–24,1)	27,4 (24,3–30,6)	21,2 (19,3–23,2)	36,5 (32,1–41,1)	23,6 (21,2–26,0)	17,8 (14,8–21,2)
≥ 12	9,8 (8,3–11,4)	18,7 (15,0–23,0)	10,3 (8,7–12,8)	24,8 (20,1–30,0)	9,2 (7,4–11,4)	10,8 (6,8–16,7)
Cor autorreferida^b^						
Branco	44,8 (39,8–50,0)	31,2 (28,7–33,8)*	45,0 (39,3–50,7)	39,5 (36,2–42,9)*	44,7 (39,3–50,2)	21,5 (19,1–24,0)*
Preto	9,8 (8,1–11,7)	39,9 (35,8–44,1)	10,6 (8,5–13,4)	48,1 (42,6–53,5)	8,8 (7,4–10,4)	28,3 (22,4–35,0)
Pardo	45,4 (41,2–49,6)	36,7 (34,5–39,0)	44,4 (39,9–48,9)	46,7 (43,6–49,6)	46,5 (41,8–51,3)	25,7 (23,2–28,4)
Renda familiar *per capita* em tercis (R$)						
1º tercil: ≤ 620	33,4 (29,8–37,2)	43,8 (41,5–46,0)*	36,0 (32,2–39,9)	51,0 (48,5–53,5)*	30,4 (26,6–34,5)	33,7 (30,1–37,6)*
2º tercil: > 620 a 1.150	33,4 (31,7–35,2)	36,2 (33,9–38,7)	33,5 (31,3–35,7)	46,2 (42,3–50,1)	33,3 (31,4–35,3)	24,6 (21,8–27,6)
3º tercil: > 1.150	33,2 (30,1–36,4)	24,7 (22,6–27,0)	30,5 (27,4–33,9)	32,8 (29,9–35,9)	36,3 (32,5–40,1)	16,7 (14,5–19,1)
Residir sozinho						
Sim	9,4 (8,3–10,6)	40,1 (36,5–43,8)*	11,3 (10,0–12,8)	42,0 (37,2–46,8)	7,1 (5,9–8,5)	36,6 (31,3–42,3)*
Não	90,6 (89,4–91,7)	34,4 (32,6–36,2)	88,7 (87,1–90,0)	44,1 (41,9–46,2)	92,9 (91,4–94,1)	23,6 (21,8–25,4)
Tempo de residência (em anos)^c^ [média (DP)]	41,0 (18,6)	41,3 (18,7)	41,8 (19,3)	41,8 (18,8)	40,2 (17,7)	40,1 (18,2)
Número de doenças crônicas^d^						
0	16,4 (15,1–17,8)	21,0 (17,8–24,5)*	10,9 (9,6–12,5)	25,8 (20,9–31,4)*	22,7 (21,0–24,5)	18,3 (14,7–22,5)*
1 a 2	48,0 (46,0–50,1)	29,5 (27,3–31,9)	44,7 (42,2–47,1)	37,1 (34,3–40,0)	51,9 (49,4–54,4)	22,0 (19,3–24,9)
≥ 3	35,6 (33,1–38,1)	47,2 (44,2–50,2)	44,4 (42,0–46,8)	54,2 (51,0–57,2)	25,4 (22,6–28,2)	33,0 (29,2–37,0)
Autoavaliação de saúde^e^						
Muito boa ou boa	45,7 (42,7–48,7)	21,5 (19,5–23,7)*	45,1 (42,0–48,1)	29,1 (26,1–32,3)*	46,4 (42,5–50,3)	13,0 (10,9–15,3)*
Regular	43,3 (41,2–45,4)	40,2 (38,4–42,4)	42,9 (40,6–45,3)	50,1 (47,1–53,1)	43,6 (40,9–46,3)	28,8 (25,9–31,9)
Ruim ou muito ruim	11,0 (9,8–12,5)	69,6 (66,2–72,7)	12,0 (10,7–13,5)	76,9 (72,3–80,9)	10,0 (8,2–12,0)	59,3 (54,3–64,1)
n (não ponderado)	7.115		4.041		3.074	

IC95%: intervalo de confiança de 95%; DP: desvio padrão; F: feminino; M: masculino.

Dados ausentes:

^a^ 39 (0,6%), F = 25 e M = 14.

^b^ 456 (6,4%), F = 262 e M = 194, com exclusão de amarelos (90, 0,99%) e indígenas (220, 2,43%).

^c^ 71 (1%), F = 61 e M = 10.

^d^ 267 (3,8%), F = 159 e M = 108.

^e^ 16 (0,2%), F = 12 e M = 4.

* p < 0,05.

A prevalência de sintomas depressivos para o total da amostra foi de 34,9% (IC95% 33,2–36,7), sendo 43,8% (IC95% 41,5–45,9) entre mulheres e 24,5% (IC95% 22,8–26,3) entre homens. Na análise univariada, para o total da amostra e por sexo, os sintomas depressivos foram associados às características sociodemográficas e de saúde (Tabela 1), bem como a percepção da vizinhança (Tabela 2) dos participantes.

**Tabela 2:** Descrição da amostra e prevalência de sintomas depressivos segundo a percepção das características da vizinhança, para o total da amostra e por sexo. Estudo Longitudinal da Saúde dos Idosos Brasileiros (ELSI-Brasil), 2015–2016.

Variável	Total		Feminino		Masculino	
	Descrição da amostra % (IC95%)	Prevalência % (IC95%)	Descrição da amostra % (IC95%)	Prevalência % (IC95%)	Descrição da amostra % (IC95%)	Prevalência % (IC95%)
Problemas de mobilidade urbana^a^						
Não	34,7 (32,0–37,4)	22,9 (20,4–25,4)*	25,7 (23,2–28,3)	30,3 (26,3–34,5)*	45,0 (41,4–48,6)	18,0 (15,4–20,8)*
Sim	65,3 (62,6–67,9)	41,5 (39,3–43,8)	74,3 (71,7–76,8)	48,9 (46,5–51,3)	55,0 (51,3–58,5)	30,0 (27,2–32,9)
Poluição sonora^b^						
Não	70,0 (68,0–71,9)	31,9 (29,9–33,9)*	70,1 (67,8–72,4)	41,2 (38,6–43,8)*	69,8 (67,5–72,0)	21,0 (18,7–23,4)*
Sim	30,0 (28,1–32,0)	41,9 (39,2–44,6)	29,9 (27,6–32,2)	50,1 (46,9–53,2)	30,2 (28,0–32,5)	32,5 (29,4–35,8)
Desordem física^c^						
Não	36,6 (33,1–40,2)	28,9 (26,5–31,3)*	36,0 (32,8–39,3)	38,9 (35,4–42,4)*	37,3 (32,9–41,8)	17,8 (15,3–20,6)*
Sim	63,4 (59,8–66,8)	38,2 (36,2–40,2)	64,0 (60,7–67,1)	46,7 (44,4–49,0)	62,7 (58,1–67,1)	28,2 (26,3–30,5)
Agradabilidade do bairro^d^						
Não	7,6 (6,5–8,8)	48,7 (44,1–53,2)*	7,1 (6,0–8,4)	60,4 (54,2–66,3)*	8,1 (6,6–9,9)	37,1 (31,3–43,2)*
Sim	92,4 (91,2–93,5)	33,7 (31,9–35,5)	92,9 (91,5–94,0)	42,7 (40,4–44,9)	91,9 (90,1–93,4)	23,6 (21,8–25,5)
Violência^e^						
Não	92,8 (91,9–93,5)	34,2 (32,4–36,1)*	93,1 (92,1–94,0)	43,2 (41,0–45,4)*	92,3 (91,1–93,4)	23,7 (21,9–25,6)*
Sim	7,2 (6,5–8,1)	43,5 (38,8–48,2)	6,9 (6,0–7,9)	53,0 (46,6–59,1)	7,7 (6,6–8,9)	33,6 (27,3–40,4)
Coesão social^f^						
Não	46,5 (43,8–49,1)	41,6 (39,6–43,5)*	47,8 (44,2–51,4)	51,2 (48,7–53,6)*	44,9 (42,2–47,6)	29,7 (26,9–32,7)*
Sim	53,5 (50,9–56,2)	29,1 (26,9–31,3)	52,2 (48,6–55,8)	37,2 (34,1–40,2)	55,1 (52,4–57,8)	20,2 (18,0–22,5)
Sensação de insegurança^g^						
Não	61,1 (57,7–64,3)	38,3 (35,6–41,1)*	61,2 (58,0–64,3)	48,0 (44,7–51,4)*	60,9 (56,5–65,2)	27,3 (23,9–30,8)*
Sim	38,9 (35,6–42,3)	32,5 (30,6–34,3)	38,8 (35,6–42,0)	41,1 (38,4–43,9)	39,1 (34,8–43,5)	22,5 (20,2–24,8)
n (não ponderado)	7.115		4.041		3.074	

IC95%: intervalo de confiança de 95%; F: feminino; M: masculino.

Dados ausentes:

^a^ 315 (4,4%); F = 202 e M = 113.

^b^ 230 (3,2%); F = 144 e M = 86.

^c^ 356 (5,0%); F = 235 e M = 121.

^d^ 401 (5,6%); F = 299 e M = 102.

^e^ 228 (3,2%); F = 143 e M = 85.

^f^ 67 (0,9%); F = 55 e M = 12.

^g^ 323 (4,5%); F = 211 e M = 112.

* p < 0,05.

Para o total da amostra, 65,3% relataram presença de problemas de mobilidade urbana; 30% poluição sonora; 63,4% de desordem física; 7,6% ausência de agradabilidade do bairro; 7,2% violência; 46,5% ausência de coesão social; 38,9% sensação de insegurança (Tabela 2).

Na análise múltipla, observou-se associação significativa para o total da amostra (T), mulheres (M), homens (H) e RP entre sintomas depressivos e problemas de mobilidade urbana (T: RP = 1,39; IC95% 1,25–1,56; M: RP = 1,37; IC95% 1,21–1,56; H: RP = 1,38; IC95% 1,15–1,64), poluição sonora (T: RP = 1,27; IC95% 1,19–1,35; M: RP = 1,18; IC95% 1,10–1,27; H: RP = 1,46; IC95% 1,26–1,70), desordem física (T: RP = 1,22; IC95% 1,12–1,33; M: RP = 1,15; IC95% 1,05–1,25; H: RP = 1,43; IC95% 1,22–1,68), ausência de agradabilidade do bairro (T: RP = 1,24; IC95% 1,22–1,39; M: RP = 1,25; IC95% 1,08–1,43; H: RP = 1,22; IC95% 1,04–1,43), violência (T: RP = 1,31; IC95% 1,17–1,45; M: RP = 1,25; IC95% 1,10–1,41; H: RP = 1,44; IC95% 1,17–1,77) e ausência de coesão social (T: RP = 1,26; IC95% 1,17–1,36; M: RP = 1,27; IC95% 1,16–1,38; H: RP = 1,26; IC95% 1,10–1,46). Foi observada diferença entre os sexos somente para sensação de insegurança, em que se evidenciou associação significativa para o total da amostra (RP = 1,13; IC95% 1,05–1,21) e entre as mulheres (RP = 1,12; IC95% 1,03–1,21), sem associação entre os homens (Tabela 3).

**Tabela 3:** Análise univariada e múltipla das associações entre percepção da vizinhança e sintomas depressivos, para o total da amostra e por sexo. Estudo Longitudinal da Saúde dos Idosos Brasileiros (ELSI-Brasil), 2015–2016.

Variável	Total		Feminino		Masculino	
	RP bruta (IC95%)	RP ajustada (IC95%)^a^	RP bruta (IC95%)	RP ajustada (IC95%)^a^	RP bruta (IC95%)	RP ajustada (IC95%)^a^
Problemas de mobilidade urbana	1,82 (1,63–2,03)	1,39 (1,25–1,56)	1,61 (1,41–1,85)	1,37 (1,21–1,56)	1,67 (1,38–2,01)	1,38 (1,15–1,64)
Poluição sonora	1,31 (1,22–1,41)	1,27 (1,19–1,35)	1,21 (1,12–1,31)	1,18 (1,10–1,27)	1,55 (1,32–1,81)	1,46 (1,26–1,70)
Desordem física	1,32 (1,23–1,43)	1,22 (1,12–1,33)	1,20 (1,10–1,32)	1,15 (1,05–1,25)	1,59 (1,35–1,87)	1,43 (1,22–1,68)
Ausência de agradabilidade do bairro	1,44 (1,31–1,59)	1,24 (1,22–1,39)	1,42 (1,27–1,59)	1,25 (1,08–1,43)	1,57 (1,32–1,86)	1,22 (1,04–1,43)
Violência	1,27 (1,14–1,41)	1,31 (1,17–1,45)	1,23 (1,09–1,38)	1,25 (1,10–1,41)	1,41 (1,14–1,75)	1,44 (1,17–1,77)
Ausência de coesão social	1,43 (1,32–1,54)	1,26 (1,17–1,36)	1,38 (1,26–1,51)	1,27 (1,16–1,38)	1,47 (1,26–1,71)	1,26 (1,10–1,46)
Sensação de insegurança	1,18 (1,09–1,27)	1,13 (1,05–1,21)	1,17 (1,06, 1,28)	1,12 (1,03–1,21)	1,21 (1,02, 1,44)	1,15 (0,98–1,35)^b^

RP: razão de prevalência; IC95%: intervalo de confiança de 95%.

^a^ Modelos ajustados pelas variáveis sociodemográficas e de saúde: faixa etária, estado civil, anos de estudo, cor autorreferida, renda familiar *per capita* em tercil, residir sozinho, tempo de residência (em anos), número de doenças crônicas (excluindo depressão, hipertensão arterial ou diabetes mellitus na gravidez) e autoavaliação de saúde.

^b^ p ≥ 0,05 (não significativo).

## DISCUSSÃO

Adultos mais velhos residentes em áreas urbanas no Brasil com sintomas depressivos relataram significativamente maior percepção de problemas de mobilidade urbana, poluição sonora, desordem física, violência, ausência de agradabilidade do bairro e de coesão social no total e nos estratos. O relato de sensação de insegurança entre aqueles com sintomas depressivos foi significativamente maior no total da amostra e entre as mulheres apenas.

Nossos achados para o total da amostra são consistentes com a literatura pregressa. A mobilidade na vizinhança é dependente da existência de ruas bem projetadas, acesso conveniente a transporte e ambiente físico agradável^14^. Nossos resultados demonstram aumento de 39% na prevalência de sintomas depressivos entre participantes que relataram perceber problemas de mobilidade urbana, como medo de cair por causa de defeitos nos passeios, preocupação com a dificuldade para subir no ônibus, metrô ou trem ou dificuldade para atravessar a rua. Um estudo realizado em um município da região sul do Brasil^15^ evidenciou que a presença de faixas de pedestre, sinais ou passarelas contribuiu para a redução da chance de sintomas depressivos em idosos (≥ 60 anos). Também, um estudo com idosos chineses (≥ 60 anos) sobre caminhabilidade^16^, classificada com base na proporção de céu visível na rua, demonstrou um aumento nos escores de depressão em decorrência da redução da caminhabilidade no bairro.

Em relação à poluição sonora, os resultados mostraram que a percepção do ruído de tráfego rodoviário associou-se ao aumento de 27% na prevalência de sintomas depressivos. Os achados corroboram com Yun et al.^17^, Lin et al.^18^ e Orban et al.^19^ No estudo de Yun et al.^17^, os autores mostraram que pessoas de diferentes idades, incluindo idosos (≥ 60 anos), que viviam em áreas com alta exposição ao ruído ambiental apresentaram aumento na chance de desenvolver sintomas depressivos leves e moderados comparadas às com menor exposição. Lin et al.^18^, em um estudo transversal de base comunitária, identificaram a associação entre a exposição ao ruído do tráfego rodoviário por 24 horas e o aumento na prevalência de depressão entre moradores de 30 a 70 anos. Orban et al.^19^, em uma coorte prospectiva de cinco anos realizada na Alemanha com participantes de 45 a 75 anos, observaram que o ruído do tráfego rodoviário residencial aumentou o risco de sintomas depressivos elevados entre adultos de meia idade e idosos.

Desordem física e agradabilidade são indicadores cujos componentes se interrelacionam, podendo ser avaliados pela presença de prédios ou casas vagos; ausência de agradabilidade para crianças, jovens e adolescentes; lixo nas ruas, calçadas; lotes vagos com lixo ou grama alta; janelas quebradas; paredes degradadas^20^. Nossos resultados demonstram que a presença de desordem física e a ausência de agradabilidade aumentam a prevalência de sintomas depressivos em 22% e 24%, respectivamente. Esses achados foram consistentes com os estudos de Pai e Kim^21^ (com população de 40 a 96 anos) e Wang et al.^22^ (com idosos ≥ 60 anos), que evidenciaram associação positiva entre desordem física e sintomas depressivos, assim como com Helbich et al.^23^, que identificaram a percepção da presença de agradabilidade negativamente correlacionada com a gravidade da depressão na população holandesa de 18 a 65 anos.

A coesão social é um recurso psicossocial protetivo contra estresse e depressão, uma vez que fornece apoio afetivo entre vizinhos^24^. Nossos resultados identificaram um aumento de 26% na prevalência de sintomas depressivos entre participantes que percebiam ausência de coesão social. Resultado convergente com Helbich et al.^23^, que evidenciaram correlação inversa entre gravidade da depressão e percepção de coesão social em estudo transversal com holandeses (18 a 65 anos), e com Ruiz et al.^25^, que demonstraram maior densidade de incidência de sintomas depressivos nos tercis mais baixos de percepção da coesão social ao longo dos 12 anos entre ingleses (≥ 50 anos).

O constructo violência remete a ter sido vítima de violência física, psicológica, roubo, furto, invasão de residência, percepção de criminalidade etc.^26,27^ Está relacionado com insegurança, que corresponde tanto à percepção de se sentir seguro na vizinhança quanto à de crimes no bairro^27^. Nossos resultados evidenciam aumento de 31% na prevalência de sintomas depressivos entre participantes que relataram ter sido vítimas de furto, roubo ou invasão da residência nos últimos 12 meses, e 12% entre as mulheres que relataram se sentir inseguras diante de crimes e violência. O aumento na prevalência de sintomas depressivos identificado é consistente com Ivey et al.^26^, em que idosos estadunidenses (≥ 65 anos) que relataram menor segurança contra crimes apresentaram maior chance de ficarem deprimidos, assim como Cândido et al.^15^, que evidenciaram menor chance de sintomas depressivos entre idosos brasileiros (≥ 60 anos) que se sentiam seguros para caminhar durante o dia e à noite no município de Balneário Arroio do Silva, no estado de Santa Catarina. Outro estudo com adultos mais velhos (50 a 74 anos) residentes na cidade de New Jersey^27^, avaliando a correlação entre taxas de violência e percepção da segurança, identificou que idosos que residiam em bairros com maiores taxas de crimes violentos e com maior preocupação em relação à segurança no bairro apresentaram maiores níveis de sintomas depressivos^27^.

A percepção de insegurança entre as mulheres pode ser efeito da percepção de maior vulnerabilidade à violência no Brasil^9^. No contexto social e cultural brasileiro, a violência contra as mulheres tem sido fenômeno crescente envolvendo agressões letais e não letais, ocorrendo em âmbito doméstico, comunitário e institucional e tendo os homens como agressores majoritários^9^. Ainda, no caso das mulheres idosas, a questão de gênero se intersecciona às alterações biológicas, econômicas e sociais decorrentes do processo de envelhecimento, que podem contribuir para o aumento da dependência nas atividades básicas diárias e, assim, maior vulnerabilidade e predisposição a ser vítima de violência praticada tanto por homens quanto por mulheres na condição de cuidadores^9^. Nosso resultado é coerente com estudo de Campos^28^, que evidenciou como mulheres idosas brasileiras de um município da região sudeste (≥ 60 anos) apresentaram maior chance de se sentirem inseguras diante da violência urbana, justamente por ser a mulher a principal vítima^28^.

Embora a depressão seja um fenômeno multifatorial, a exposição a situações estressantes pode estar no caminho causal^29^. Custrona et al.^5^ analisam que o estresse desempenha importante papel explicativo na associação entre características da vizinhança e o desenvolvimento da depressão. Os autores propõem um modelo explicativo que considera a vizinhança como produtora de níveis diários de estresse, advindos de possíveis aumentos na vulnerabilidade dos moradores diante de eventos negativos e perda de laços sociais, levando à depressão. As respostas ao estresse podem desgastar o corpo e produzir modificações patológicas, como diminuição dos neurônios do hipocampo, redução da neurogênese e problemas cognitivos em razão do excesso de cortisol produzido^29^.

Nesse sentido, morar em um ambiente com desordem física pode produzir uma sensação de desamparo e gerar estresse nos moradores^5^. Também, a poluição sonora pode contribuir para doses diárias de estresse, agravando os impactos ambientais sobre o bem-estar psicológico. Ainda, adultos mais velhos com problemas de mobilidade urbana que vivem em vizinhanças com barreiras ambientais experimentam a redução nas possibilidades de interação social, contribuindo para o isolamento e favorecendo o aparecimento de sintomas depressivos^30^. Por fim, a violência e a insegurança atuam como estressores ambientais^16,27^, o que gera uma sensação constante de perigo, contribuindo também para o isolamento social.

Ademais, a coesão social amortece o estresse decorrente da percepção negativa da vizinhança e, assim, o impacto sobre a saúde mental dos moradores, configurando-se como potencial recurso protetor do desenvolvimento da depressão pela redução do isolamento e aumento da interação social^24^. Portanto, a ausência desse atributo colabora para o aumento do estresse da vizinhança e seus efeitos sobre o aumento na prevalência de sintomas depressivos.

Nosso estudo apresenta limitações que devem ser mencionadas. Primeiro, a característica autorreferida da escala utilizada para mensurar o desfecho pode estar sujeita ao viés do respondente. Os participantes podem, por exemplo, minimizar seus sintomas depressivos por desconhecimento ou mediante questões culturais. Além disso, o caráter transversal do estudo requer cautela na interpretação dos resultados em razão de possível viés de causalidade reversa. No entanto, nossos achados foram consistentes com estudos transversais e longitudinais anteriores^15^. Cabe destacar que este estudo apresenta pontos fortes, como amostra robusta, nacionalmente representativa de adultos brasileiros com 50 anos ou mais, em um país de média renda.

Ao investigar diferentes domínios da percepção da vizinhança (ambiente físico e social), os resultados revelam uma estreita relação entre sintomas depressivos em adultos mais velhos brasileiros e espaço urbano. Nossos achados indicam que a presença de problemas de mobilidade urbana, poluição sonora, desordem física, violência, ausência de agradabilidade do bairro e coesão social aumentaram a prevalência de sintomas depressivos independentemente do gênero. A sensação de insegurança se associou ao aumento na prevalência de sintomas depressivos somente entre as mulheres, o que pode estar relacionado ao cenário brasileiro de violência de gênero e seus desdobramentos na produção de vulnerabilidades específicas no processo de envelhecimento das mulheres.

Os resultados apontam para a importância de uma ação intersetorial entre as políticas de saúde, mobilidade urbana, segurança pública e planejamento e organização das cidades, com foco na promoção à saúde mental de todos os moradores, sobretudo mulheres e homens mais velhos moradores em áreas urbanas. Assim, reduzir a percepção negativa de adultos mais velhos brasileiros sobre a vizinhança poderá refletir na melhora do bem-estar e saúde mental na velhice, contribuindo para a redução na prevalência de sintomas depressivos.

## Data Availability

Os dados da pesquisa estão disponíveis mediante registro e solicitação através do link https://elsi.cpqrr.fiocruz.br/data-access/
